# The complete chloroplast genome of *Meeboldia yunnanensis* (Apiaceae)

**DOI:** 10.1080/23802359.2019.1644217

**Published:** 2019-11-21

**Authors:** Wei Gou, Xian-Lin Guo, Yan Yu, Song-Dong Zhou, Xing-Jin He

**Affiliations:** Key Laboratory of Bio-Resources and Eco-Environment of Ministry of Education, College of Life Sciences, Sichuan University, Chengdu, Sichuan, People’s Republic of China

**Keywords:** *Meeboldia yunnanensis*, complete chloroplast genome, phylogenetic analysis

## Abstract

*Meeboldia yunnanensis* Wolff (Apiaceae) is a perennial species naturally distributed in Yunnan and Xizang. The complete chloroplast genome sequence of *M. yunnanensis* was generated by *de novo* assembly using whole-genome next-generation sequencing data. The complete chloroplast genome of *M. yunnanensis* was 154,865 bp in total sequence length and divided into four distinct regions: small single-copy region (17,370 bp), large single-copy region (84,641 bp), and a pair of inverted repeat regions (26,427 bp). The genome annotation displayed a total of 130 genes, including 85 protein-coding genes, 37 tRNA genes, and 8 rRNA genes. Phylogenetic analysis with the reported chloroplast genomes revealed that *M. yunnanensis* has close relationship to *Pterygopleurum neurophyllum*.

*Meeboldia yunnanensis* Wolff (Apiaceae) is a perennial herb, which is naturally distributed in Yunnan and Xizang. It grows sparsely on mountain slopes, grassy places, and rock crevices with an altitude of 2000–3500 m (She and Watson 2005). The plants are used in Yunnan as a regional substitute for the medicine ‘gao ben’ (*Ligusticum sinense* and *Ligusticum jeholense*) (She and Watson 2005). There are three species in *Meeboldia* H. Wolff which distribute in Sino–Himalayan region and two species (one endemic) in China (She and Watson 2005). Here, we reported the complete chloroplast (cp) genome of *M. yunnanensis*, and deposited the annotated cp genome into GenBank with the accession number MK993275.

Fresh leaves of *M. yunnanensis* was collected from Haba Village (27°22′24″N 100°8′12″E), Yunnan Province, China. Voucher specimens were deposited in SZ (Sichuan University Herbarium) and the voucher number of specimen is G1807190801. The genomic DNA was extracted following the modified CTAB method from the dry and healthy leaves (Doyle and Doyle 1987). The isolated genomic was manufactured to average 350 bp paired-end(PE) library using Illumina NovaSeq6000 platform (Illumina, San Diego, CA), and sequenced by Illumina genome analyzer (Hiseq PE150). The filtered reads were assembled using the program NOVOPlasty (Dierckxsens et al. 2017) with the complete chloroplast genome of its close relative *Pterygopleurum neurophyllum* (Maxim.) Kitag. as the reference (GenBank accession no. NC_033345). The assembled chloroplast genome was annotated using Geneious 11.0.4 and corrected manually (Kearse et al. 2012). The complete chloroplast genome of nine species was aligned using MAFFT (Katoh et al. 2002). Nine species of Apiaceae were employed to build the maximum-likelihood (ML) tree using RaxML (Stamatakis 2006) with 1000 bootstrap replicates. The chloroplast genome of *M. yunnanensis* was 154,865 bp in length (GenBank accession no. MK993275), containing a large single-copy region (LSC) of 84,641 bp, a small single-copy region (SSC) of 17,370 bp, and a pair of inverted repeat (IR) regions of 26,427 bp. Genome annotation predicted 127 genes, including 82 protein-coding genes, 37 tRNA genes, and 8 rRNA genes. The overall GC-content of the whole plastome was 37.6%, with the corresponding values in the LSC, SSC, and IR regions were 35.7, 31.0, and 42.7%, respectively.

With released complete cp DNA of Apiaceae, phylogenetic analysis suggested that *M. yunnanensis* is close to *Pterygopleurum neurophyllum* (Figure 1), which was consistent with a previous study (Zhou et al. 2009). This complete cp genome can provide a genomic resource and contribute to constructing phylogenetic relationships and evolutionary studies among the genus *Meeboldia* H. Wolff and relative groups.

**Figure 1.**
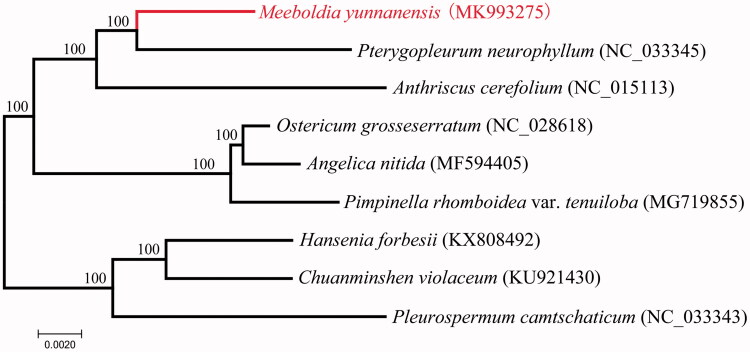
ML phylogenetic tree of *M. yunnanensis* with eight species of Apiaceae was constructed by chloroplast genome sequences. Numbers on the nodes are bootstrap values from 1000 replicates.
